# Transcriptomic analysis reveals diverse gene expression changes in airway macrophages during experimental allergic airway disease

**DOI:** 10.12688/wellcomeopenres.15875.2

**Published:** 2020-06-22

**Authors:** William J. Branchett, Anne O'Garra, Clare M. Lloyd

**Affiliations:** 1 National Heart and Lung Institute, Imperial College London, London, SW7 2AZ, UK; 2 Asthma UK Centre in Allergic Mechanisms of Asthma, Imperial College London, London, W2 1NY, UK; 3 Laboratory of Immunoregulation and Infection, The Francis Crick Institute, London, NW1 1AT, UK

**Keywords:** Macrophage, lung, airway, allergy, asthma, transcriptomics, house dust mite, mouse models

## Abstract

**Background: **Airway macrophages (AMs) are the most abundant leukocytes in the healthy airway lumen and have a highly specialised but plastic phenotype that is governed by signals in the local microenvironment. AMs are thought to maintain immunological homeostasis in the steady state, but have also been implicated in the pathogenesis of allergic airway disease (AAD). In this study, we aimed to better understand these potentially contrasting AM functions using transcriptomic analysis.

**Methods: **Bulk RNA sequencing was performed on AMs (CD11c ^+^ Siglec F ^+^ CD64 ^+^ CD45 ^+^ SSC ^hi^) flow cytometry sorted from C57BL/6 mice during experimental AAD driven by repeated house dust mite inhalation (AMs ^HDM^), compared to control AMs from non-allergic mice. Differentially expressed genes were further analysed by hierarchical clustering and biological pathway analysis.

**Results: **AMs ^HDM ^showed increased expression of genes associated with antigen presentation, inflammatory cell recruitment and tissue repair, including several chemokine and matrix metalloproteinase genes. This was accompanied by increased expression of mitochondrial electron transport chain subunit genes and the retinoic acid biosynthetic enzyme gene *Raldh2*. Conversely, AMs ^HDM ^displayed decreased expression of a number of cell cycle genes, genes related to cytoskeletal functions and a subset of genes implicated in antimicrobial innate immunity, such as *Tlr5*, *Il18* and *Tnf*. Differential gene expression in AMs ^HDM ^was consistent with upstream effects of the cytokines IL-4 and IFN-γ, both of which were present at increased concentrations in lung tissue after HDM treatment.

**Conclusions: **These data highlight diverse gene expression changes in the total AM population in a clinically relevant mouse model of AAD, collectively suggestive of contributions to inflammation and tissue repair/remodelling, but with decreases in certain steady state cellular and immunological functions.

## Introduction

Immunity must be tightly regulated in the respiratory tract, to avoid overexuberant immune responses in delicate lung tissue without compromising immunity to airborne pathogens. Allergic asthma is a disease of chronic pulmonary inflammation and airway remodelling that involves pro-inflammatory functions of diverse leukocytes of the innate and adaptive immune response, typically driven by type 2 immunity ^1^. In addition, deficiencies in immune regulatory mechanisms have been associated with the incidence and severity of asthma and experimental allergic airway disease (AAD) phenotypes ^2^. Characterising the cellular and molecular determinants that control the balance between pro- and anti-inflammatory mechanisms in AAD is therefore central to understanding the immunological basis of allergic asthma.

Airway macrophages (AMs) are the most abundant leukocytes in the healthy airway lumen ^3^ and are essential for pulmonary surfactant metabolism ^4, 5^. They differentiate from foetal precursors in the first week of life and can self-renew during adulthood independently of circulating monocytes ^6, 7^, although replacement from bone marrow-derived monocytes is observed with advancing age ^8, 9^. AMs have a distinctive transcriptional and cell surface marker profile that is imprinted by the airway microenvironment, including high surface expression of the integrin CD11c and sialic acid-binding lectin (Siglec) F ^6, 10^. AMs are relatively poor activators of adaptive immunity compared to other macrophage subsets ^11^ and their depletion *in vivo* results in enhanced pulmonary immune responses ^12, 13^, leading to the view that AMs act to maintain pulmonary immune homeostasis in health ^14^. AMs also show a muted response to the type 2 cytokine IL-4 when delivered to naïve mice, compared to interstitial macrophages (IMs) that exist outside of the airspaces of the lung ^15^, consistent with a relative hyporesponsiveness of AMs to a number of activating stimuli in the steady state. However, AMs are also crucial drivers of early inflammatory responses to pathogens, including respiratory syncytial virus ^16, 17^, and are important for innate protection from respiratory bacterial infections ^18^.

Both pro- and anti-inflammatory functions have also been suggested for AMs during AAD, based in part on mechanistic experiments in animal models. Depletion of AMs by delivery of cytotoxic clodronate-loaded liposome to the airways of mice augments pulmonary inflammatory responses to allergens ^19– 21^ and delays resolution of elevated Th2 cell numbers following cessation of allergen exposure ^21^, consistent with an anti-inflammatory, pro-resolving role of AMs in the context of aeroallergen exposure. Moreover, adoptive transfer of antigen-pulsed AMs to naïve mice limited subsequent allergic sensitisation to the same antigen, concomitant with increased generation of FoxP3 ^+^ Tregs ^22^. However, adoptive transfer of AMs lacking the classical macrophage activation transcription factor IRF5 into naïve mice resulted in an increased type 2 immune response upon subsequent allergen inhalation, indicating that AMs can promote inflammation, rather than tolerance, when dysregulated ^23^. Accordingly, AMs expressed high levels of the major Th1 cell attracting chemokines CXCL9 and CXCL10 in a mouse model of Th1 ^hi^ AAD ^24^. AMs during AAD are therefore likely to differ markedly in phenotype and function from those at steady state, likely reflecting the inherent plasticity of macrophage phenotypes, particularly when faced with a complex and dynamic set of polarising signals such as during inflammatory responses ^25^. Indeed, expression of molecules associated with allergic inflammation and airway remodelling has been observed in AMs during asthma and experimental AAD ^26– 29^.

The complex and potentially conflicting pro- and anti-inflammatory functions of AMs during AAD warrant further studies of the molecular features of AMs in the allergic lung, to better understand their likely *in vivo* roles and the signals controlling these processes. Herein, we describe results of bulk RNA sequencing (RNA-seq) on AMs sorted from mice during a well-established model of AAD driven by inhalation of the clinically relevant aeroallergen house dust mite (HDM), against those from allergen-naive control mice. AMs from HDM-treated mice, as compared to controls, showed decreased expression of genes related to certain steady state behaviours (cell cycle, cytoskeletal functions, a subset of pattern recognition receptors and innate cytokines), but marked increases in genes associated with inflammatory cell recruitment, extracellular matrix (ECM) remodelling, oxidative metabolism and generation of the immunoregulatory molecule, retinoic acid. Gene expression signatures of AMs during HDM-driven AAD were suggestive of regulation by multiple upstream cytokine signals, including IFN-γ and IL-4. This work provides insights into potential phenotypic changes to AMs during AAD using an unprecedented RNA-seq approach, which will aid understanding of their protective and pathogenic functions *in vivo*. 

## Methods

Additional information on key materials is presented in Table 1.

**Table 1.** Key reagent and material details.

Resource	Source	Identifier
Experimental models: organisms and strains		
C57BL/6J mice	Charles River Laboratories, UK	N/A
**Biological Samples**		
House dust mite extract (whole crushed mite bodies)	Greer Laboratories	XPB70D3A25
**Antibodies**		
PERCP/Cy5.5 CD45 (clone 30-F11) Working concentration 1 in 200	BioLegend	RRID:AB_893340
Brilliant Violet 421 CD64 (clone X54-5/7.1) Working concentration 1 in 50	BioLegend	RRID:AB_2562694
Brilliant Violet 605 CD11b (clone M1/70) Working concentration 1 in 200	BioLegend	RRID:AB_2565431
APC/Cy7 CD11c (clone N418) Working concentration 1 in 100	Biolegend	RRID:AB_830649
PE Siglec F (clone E50-2440) Working concentration 1 in 100	BD Biosciences	RRID:AB_394341
Purified anti-mouse CD16/CD32 (Mouse Fc Block™; clone 2.4G2) Working concentration 1 in 100	BD Biosciences	RRID:AB_394656
Purified IL-4 capture antibody (clone 1B11)	BD Biosciences	RRID:AB_397187
Biotinylated IL-4 detection antibody (clone BVD6-24G2)	BD Biosciences	RRID:AB_395362
Recombinant murine IL-4 ELISA standard	BD Biosciences	Cat: 550067
**Critical commercial assays and kits**		
To-Pro3 Iodide Viability Dye	Thermo Fisher	Cat: T3605
IFN-γ Mouse ELISA Kit	Thermo Fisher (eBioscience)	Cat: 88-7314-88
RNeasy Plus Micro kit	QIAGEN	Cat: 74034
TruSeq Stranded mRNA HT	Illumina	Cat: 20020595
**Software**		
FlowJo v9	Tree Star, Inc	N/A
Prism v7	GraphPad Software, Inc	N/A
StrandNGS v3.3	Strand Life Sciences	Build 23891
DESeq	Anders & Huber ^30^	N/A
ClustVis	Metsalu & Vilo ^31^	N/A
Ingenuity pathway analysis	QIAGEN	N/A

### Mice

A total of 10 mice were used in this study. Female C57BL/6J mice were purchased from Charles River Laboratories and housed at Imperial College London in individually ventilated cages with wood shaving bedding throughout experiments. Mice had *ad libitum* access to food and water and were maintained under a 12 hour light/dark cycle. Mice were housed under specific pathogen free conditions in cages of 5. Experiments were initiated with mice at 7–9 weeks of age and weighing approximately 20 g. At the time of experiment initiation, mice had not undergone any prior treatments and had been housed for 1 week in the Imperial College London facility to acclimatise. All work with live animals was performed according the Animals (Scientific Procedures) Act 1986 and local guidelines at Imperial College London.

### Induction of allergic airway disease

Individual cages of mice were assigned to either HDM or PBS control groups prior to inspection or unique identification by the researcher performing procedures, to minimise the potential for subjective bias. A target group size of 5 was determined based on a previous study in sorted macrophages during type 2 immune responses ^32^ and our own extensive experience with the HDM mouse model. However, we were unable to obtain sufficient AM RNA from one mouse in the HDM group, resulting in final group sizes of N=5 for PBS treatment and N=4 for HDM treatment. The experimental unit for all analysis was 1 individual mouse. A total of 25 μg of HDM extract (Greer Laboratories) in 25 μl sterile PBS (Gibco, Thermo Fisher) or 25 μl PBS control, were delivered intranasally to mice under brief isoflurane anaesthesia (3.5% isoflurane with oxygen as the carrier gas) in an inhalation chamber 5 days per week with a 2 day break, for 3 weeks in total, as described previously ^33^. The anaesthetic protocol was selected to be minimally invasive and cause deep nasal breathing to promote allergen inhalation. Allergen challenges were performed during light hours, between 9 am and 12.30 pm. PBS treatments were performed first, immediately followed by HDM treatments. Mice were euthanised and analysed 24 hours after the final intranasal challenge. PBS-treated mice were euthanised and dissected immediately before HDM-treated mice, with all dissections conducted within an approximately 2 hour period.

### Bronchoalveolar lavage and lung tissue

Mice were euthanised by intraperitoneal overdose with 150 μl of 200 mg/ml pentobarbital (approximately 1500 mg/kg), followed by terminal exsanguination via cardiac puncture to avoid damage to the lungs and trachea. Mice were tracheotomised with 19 G cannulas and lungs lavaged a total of 7 times with separate 1 ml volumes of 10 mM EDTA in PBS (both Gibco, Thermo Fisher), leaving the fourth volume of EDTA in the lungs for 5 minutes to promote detachment of cells adherent to the pulmonary epithelium. Lavage volumes were pooled and centrifuged at 300 *g* for 5 minutes at 4°C to obtain cell pellets. Total BAL cell counts were determined by staining cells with 0.02 % crystal violet (Sigma-Aldrich) in 700 mM acetic acid in PBS and counting manually using haemocytometer slides.

Post-lavage lungs were removed and individual right lung lobes dissected and flash frozen in liquid nitrogen. Right middle lung lobes were homogenised for protein analysis using a FastPrep-24 bead homogeniser and Lysing Matrix D (both MP Biomedicals) at a concentration of 50 mg tissue/ml of Hank’s buffered salt saline (Gibco, Thermo Fisher) without calcium or magnesium, supplemented with 1 cOmplete Protease Inhibitor cocktail tablet (Roche) per 50 ml solution.

### Flow cytometry sorting

BAL cell pellets were washed once in flow cytometry grade PBS (Thermo Fisher), before suspending in sort buffer (0.1 % foetal bovine serum [Sigma Aldrich], 1 mM EDTA and 25 mM HEPES [Gibco, Thermo Fisher] in flow cytometry grade PBS) containing fluorescently-conjugated antibodies to cell surface markers as detailed in Table 1, in the presence of 5 μg/ml Mouse Fc Block (BD Biosciences) and incubating for 20 minutes at 4°C in the dark. Cells were then washed twice and resuspended in sort buffer. Cells were filtered through 70-μm filter mesh and stained with 20 nM ToPro3 iodide viability dye (Invitrogen, Thermo Fisher) immediately prior to sorting. AMs were sorted as ToPro3 ^-^ CD45 ^+^ FSC ^hi^ SSC ^hi^ Siglec F ^+^ CD11c ^+^ CD64 ^+^ on a BD FACSAria using a 100-μm nozzle and purity sort settings. Purity checks showed 97.0% and 90.4% for PBS and HDM groups, respectively.

Numbers of AMs and eosinophils (ToPro3 ^-^ CD45 ^+^ Siglec F ^+^ CD64) in BAL were determined by analysing data from 10 ^5^ events acquired during sorts, analysed using FlowJo software (TreeStar) and multiplied by total crystal violet cell counts.

### Enzyme-linked immunosorbent substrate assays of lung cytokines

Cytokines were detected using standardised sandwich ELISAs on lung tissue using reagents as detailed in Table 1. IFN-γ was quantified using the Ready-Set-Go mouse IFN-γ ELISA kit (eBioscience, Thermo Fisher), as per manufacturer’s instructions. IL-4 was measured by coating plates with 2 μg/ml IL-4 capture antibody in 0.1 M NaHPO _4_ overnight at 4°C, before blocking for 2 hours in 1% BSA, incubating with lung homogenate samples and recombinant IL-4 standards overnight at 4°C and detecting by sequentially incubating with biotinylated IL-4 detection antibody for 1 hour and streptavidin-horse radish peroxidase for 3 minutes. Samples, standards, detection antibody and streptavidin-HRP were diluted in 1% BSA + 0.05% Tween-20 in PBS. Plates were washed with 0.05% Tween-20 in PBS between incubations.

All ELISAs were developed after streptavidin-HRP incubation using TMB substrate (eBioscience, Thermo Fisher) and reactions stopped using an equal volume of 0.18 M sulphuric acid. Results were determined by measuring absorbance at 450 nm using a Tecan Sunrise plate reader and Magellan software and unknown values interpolated into standard curves in Prism v7 using the second order polynomial equation.

### RNA extraction

Cells were pelleted and lysed in RLT buffer supplemented with 1% β-mercaptoethanol within 30 minutes of sorting, passing lysates through QIAShredder columns (QIAGEN) as per manufacturer’s instructions. RNA was extracted from lysates using the RNeasy Micro Plus kit (QIAGEN) according to manufacturer’s instructions. RNA quantity and integrity was determined using the 2200 Tapestation and RNA Screentape reagents (Agilent). All RNA integrity (RIN) values were ≥9.5.

### Library preparation and RNA sequencing

A total of 200 ng of total RNA from each sample was converted to mRNA sequencing libraries using the Illumina TruSeq Stranded mRNA HT kit, following the standard HT protocol and using 15 PCR amplification cycles. Library cDNA concentrations were determined using QuantiFluor double-stranded DNA system and GloMax plate reader (Promega) and fragment sizes determined using the Agilent 2100 Bioanalyzer, allowing library concentrations to be normalised to 10 nM. Libraries were pooled and run within single lanes of the Illumina HiSeq 4000, obtaining 75 base pair (bp) paired end reads.

### RNA sequencing analysis pipeline

Demultiplexed sequence files were subjected to FASTQC analysis (Babraham Institute), removing reads with mean phred scores of ≤20. Filtered reads were aligned to the UCSC Mm10 reference genome and transcriptome using the COBWeb aligner (based on the Burrows-Wheeler transform) within the StrandNGS software package (version 3.3, Strand LS), using the developer’s recommended settings. Reads required minimum match length of 25 bp, sequence identity of ≥90% and a maximum of 5% gaps in overlap to map to a genomic location and only the best matching genomic location for each read was considered. Reads were filtered if they mapped to five or more different genomic locations, if they aligned to mouse ribosomal RNA or UniVec sequences or if both paired reads did not align to the same genomic location. In total, >92% of all reads in each sample were successfully aligned using these criteria. Alternative open access aligner tools also based on the Burrows-Wheeler transform include Burrows-Wheeler Aligner and Bowtie 2. Open access tools such as clean_reads can be used to filter Univec sequences from FASTQ files prior to alignment and ribosomal RNA contamination can be detected and filtered in post-alignment BAM files using RSeQC.

Aligned reads were assigned to genes and transcripts according to the Refseq (2013) annotations within StrandNGS, with each read contributing a score of up to 1.0 to an exon, based on the proportion of the read length mapping to the exon. Transcripts were quantified from aligned reads using the DESeq method ^31^ within StrandNGS. DESeq is an open access Bioconductor package that can be run outside of StrandNGS. A lower normalised expression limit of 8, required in ≥75% of samples in either experimental group, was imposed to filter low abundance and potential non-AM contaminant transcripts. Principal component analysis (PCA) was performed on all filtered genes using ClustVis ^34^, using single value decomposition to calculate the two major principal components and visualise data after unit variance scaling of normalised expression values. Differential expression analysis was performed on filtered genes using a moderated unpaired *t*-test within StrandNGS, using the Benjamini-Hochberg method to adjust the *p* value for the false discovery rate (FDR). Comparable differential expression analysis can be performed on a matrix of genes and normalised expression values using the limma Bioconductor package. Unless otherwise stated, differentially expressed genes refer to genes with a fold change ≥2 and an FDR-adjusted *p*-value <0.01 in AMs from HDM-treated mice compared to AMs from PBS controls.

### Hierarchical clustering

Clustering of unscaled differentially-expressed genes was performed using Ward’s minimum variance method within StrandNGS, clustering on Euclidean distance between data points. A list of all differentially-expressed genes and their expression clusters is included as extended data for this article. Clustering of smaller selected gene sets was performed using ClustVis after unit variance scaling expression values for each gene, again clustering using Ward’s method and based on Euclidean distance.

### Ingenuity Pathway Analysis

Selected gene lists and their associated normalised expression and fold change values were uploaded to Ingenuity Pathway Analysis (IPA; QIAGEN, Spring 2017 release) and core analyses performed, restricting the considered interactions to those observed experimentally in *Mus musculus*. Open access alternative tools for pathway analysis include Reactome and PANTHER. Canonical pathways were determined to be highly enriched within gene lists if they had a positive or neutral/not applicable *z*-score for activation and a Fisher’s exact *p*-value of <0.01 and up to 15 of the top enriched pathways reported for each gene set (ranked on *p* value). Representative genes from highly enriched pathways in gene lists were manually selected on the basis of a combination of i) greatest fold difference between experimental groups, ii) involvement in >1 of the highly enriched pathways and iii) a well-established role in the pathway/biological process of interest. Where relevant, individual canonical pathways were visualised using the Path Designer tool and fold change values overlaid as colours.

Upstream regulator analysis was performed on total differentially expressed genes and results filtered to limit to cytokines and growth factors with a positive activation *z*-score. Genes with differential expression patterns consistent with activation of upstream cytokines of interest were visualised using the Path Designer tool, adding known regulatory interactions at the level of expression or transcription between genes and the upstream cytokine of interest to generate a network and overlaying fold change values as colours.

### Gene set enrichment analysis

Lists of differentially expressed genes in *in vitro*-stimulated macrophages were obtained from the publicly available GEO records associated with published microarray data ^35^ and used to generate .grp gene set files, which were tested simultaneously for enrichment in a .gct file of normalised expression values of all genes filtered on the lower expression threshold using the standard gene set enrichment analysis protocol ^36^. FDR-corrected *q*-values and normalised enrichment scores are presented. Gene lists used for GSEA are included in the Open Science Framework repository entry supporting this work (see *Extended data*).

### Other statistical analysis

Cell counts and lung cytokine concentrations were compared between PBS and HDM groups using two-tailed, unpaired, Student’s t-tests in GraphPad Prism v7. A *p*-value of <0.05 was considered statistically significant using the notation: *, *p* < 0.05; **, *p* < 0.01, ***, *p* < 0.001 and ns, not significant.

## Results

### Differential expression of discrete clusters of genes in airway macrophages during house dust mite-driven allergic airway disease

**Figure 1.**
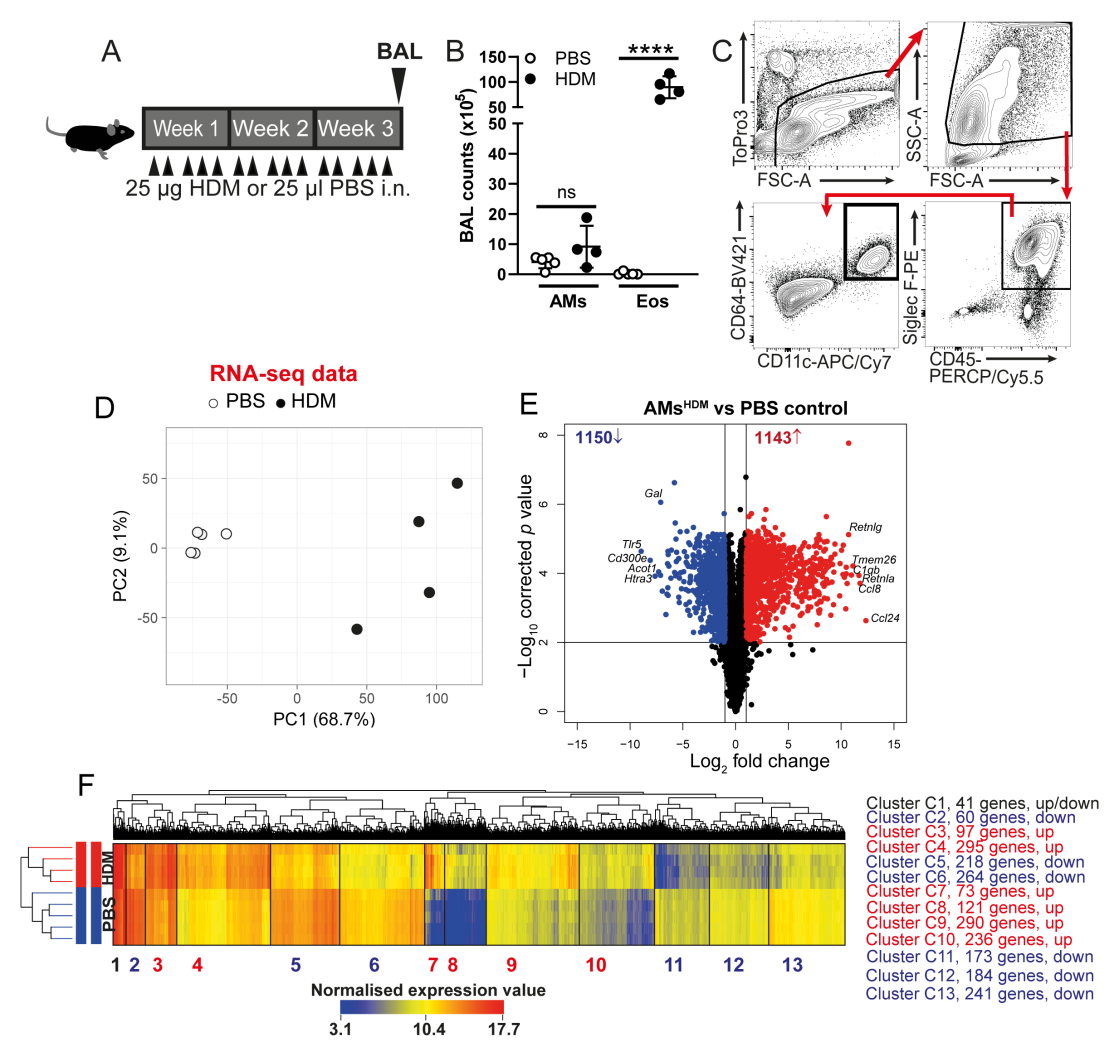
Identification and clustering of differentially expressed AM genes in the mouse HDM model. ( **A**) Schematic of the HDM AAD model, with arrows indicating individual intranasal challenges. ( **B**) Absolute counts of AMs and eosinophils (eos) in BAL as determined by flow cytometry. Means, standard deviations and individual replicate values are shown. ( **C**) Gating strategy for flow cytometry sorting of total AMs from BAL. Representative plots from a HDM-treated mouse are shown. ( **D**) Principal component analysis based on all genes (filtered on minimum expression) in AMs ^HDM ^and control AMs from PBS-treated mice. ( **E**) Volcano plot showing the results of differential expression analysis. ( **F**) Hierarchical clustering or differentially expressed genes with major clusters (3 ^rd^ tier of dendrogram) highlighted. Cluster characteristics are shown to the right. N=5 mice, PBS; N=4 mice, HDM.

To examine gene expression changes in AMs during AAD driven by a clinically-relevant aeroallergen, mice were repeatedly exposed to inhaled HDM extract for 3 weeks ( Figure 1A), a model shown previously in our laboratory to induce the hallmark features of allergic asthma, including elevated serum IgE, airway hyperresponsiveness, production of type 2 cytokines and eosinophilic pulmonary inflammation ^33^. Accordingly, bronchoalveolar lavage (BAL) from HDM-treated mice contained increased numbers of eosinophils compared to PBS-treated non-allergic controls, without a concomitant increase in AM numbers ( Figure 1B). CD11c ^+^ Siglec F ^+^ CD64 ^+^ CD45 ^+^ SSC ^hi^ AMs were flow cytometry sorted from BAL ( Figure 1C) of HDM- and PBS-treated mice, before rapidly lysing cells for RNA extraction and RNA-seq (full details in *Methods*). See *Underlying data* for flow cytometry output values ^37^.

Visualisation of overall similarities between AM transcriptomes by PCA showed AMs from HDM-treated mice (hereafter AMs ^HDM^) and from PBS-treated mice (hereafter ‘control AMs’) to differ substantially in gene expression, with marked separation between the groups in PC1 (68.7% of variance; Figure 1D). Some variability with the HDM group was observed, particularly in PC2 (9.1% of variance; Figure 1D). Consistent with their separation by PCA, more than 2000 differentially expressed genes (FDR-adjusted *p* < 0.01; fold change ≥ 2) were observed in AMs ^HDM^ compared to control AMs, with roughly equal numbers of up- and down-regulated genes ( Figure 1E). The most substantially downregulated genes included the pattern recognition receptor *Tlr5*, while the genes with the greatest fold increase were those associated with IL-4-dependent alternative macrophage activation *in vitro* and *in vivo*, including the chemokine *Ccl24* and resistin-like molecules *Retnla* and *Retnlg* ^32, 36^ ( Figure 1E).

To facilitate biological interpretation of the large number of genes with altered expression in AMs ^HDM^, all DEGs were hierarchically clustered, reasoning that functionally related genes would show similar expression patterns between groups. Clustering was performed on normalised expression values without row scaling, so as to group DEGs based on both their steady state expression in control AMs and the direction and extent of changes in AMs ^HDM^ ( Figure 1F). A total of 13 major clusters were identified, 12 of which consisted exclusively of either increased (6 clusters) or decreased (6 clusters) genes ( Figure 1F). The remaining cluster at this level, C1, consisted of 41 genes with very high expression in control AMs that contained 2 sub-clusters, a larger set of 31 upregulated genes and a smaller set of 10 down regulated genes ( Figure 1F and Table 2). Consistent with their high steady state expression, genes in C1 were generally those associated with the homeostatic phenotype and functions of AMs, including several phagosomal cathepsin genes ( *Ctsb*, *Ctsk*, *Ctss* and *Ctsv*) and *Itgax* (CD11c) among upregulated genes and the apoptotic cell uptake receptor *Axl* among downregulated genes ( Table 2), consistent with the reported downregulation of this molecule on AMs during allergic airway inflammation ^38^.

**Table 2.** Details of AM ^HDM^ differentially expressed gene cluster 1. List of all DEGs within cluster C1, separated into the major sub-cluster of genes increased in AMs ^HDM^ (red) and the smaller sub-cluster decreased in AMs ^HDM ^ (blue). Absolute fold changes in AMs ^HDM^ relative to control AMs are shown.

C1 increased in AMs ^HDM^		C1 decreased in AMs ^HDM^	
Gene	Fold change	Gene	Fold change
*Spp1*	34.393	*Iqgap1*	-2.108
*Ctsv*	17.872	*Lrp1*	-2.123
*Lgmn*	8.59	*Abcg1*	-2.199
*Mmp19*	8.449	*Cd164*	-2.263
*Tgm2*	7.455	*Ahnak*	-2.297
*Cd36*	6.252	*Cd9*	-2.308
*Chil3*	5.865	*Myh9*	-2.374
*Ctsb*	5.855	*Axl*	-2.602
*Ctsk*	5.304	*Sort1*	-3.142
*Ccl6*	4.495	*Flna*	-3.843
*Psap*	4.359		
*B2m*	3.684		
*H2-K1*	3.595		
*Clec7a*	3.551		
*C3*	3.53		
*Ctsz*	3.494		
*Fn1*	3.435		
*Npc2*	3.057		
*Ctss*	3.022		
*Anxa1*	3.02		
*Ifi30*	2.987		
*Lgals3*	2.965		
*Hspa5*	2.819		
*H2-D1*	2.538		
*Pdia3*	2.307		
*Hsp90b1*	2.283		
*Slc7a2*	2.227		
*Atp6v1b2*	2.206		
*Itgax*	2.106		
*P4hb*	2.06		
*Lipa*	2.028		

Hierarchical clustering of DEGs in AMs ^HDM^ therefore reveals discrete patterns of gene expression in AMs during AAD, with C1 highlighting highly expressed core AM genes that undergo expression changes following HDM treatment.

### AMs show decreased expression of genes related to cell cycle, cytoskeletal interactions and related intracellular signalling during HDM-driven AAD

To better understand the observed changes in gene expression in AMs ^HDM^, literature-guided functional annotation of DEG clusters was performed using Ingenuity Pathway Analysis (IPA). Highly enriched pathways were observed in all gene clusters except for C10 (236 genes, upregulated) and C11 (173 genes, downregulated).

**Figure 2.**
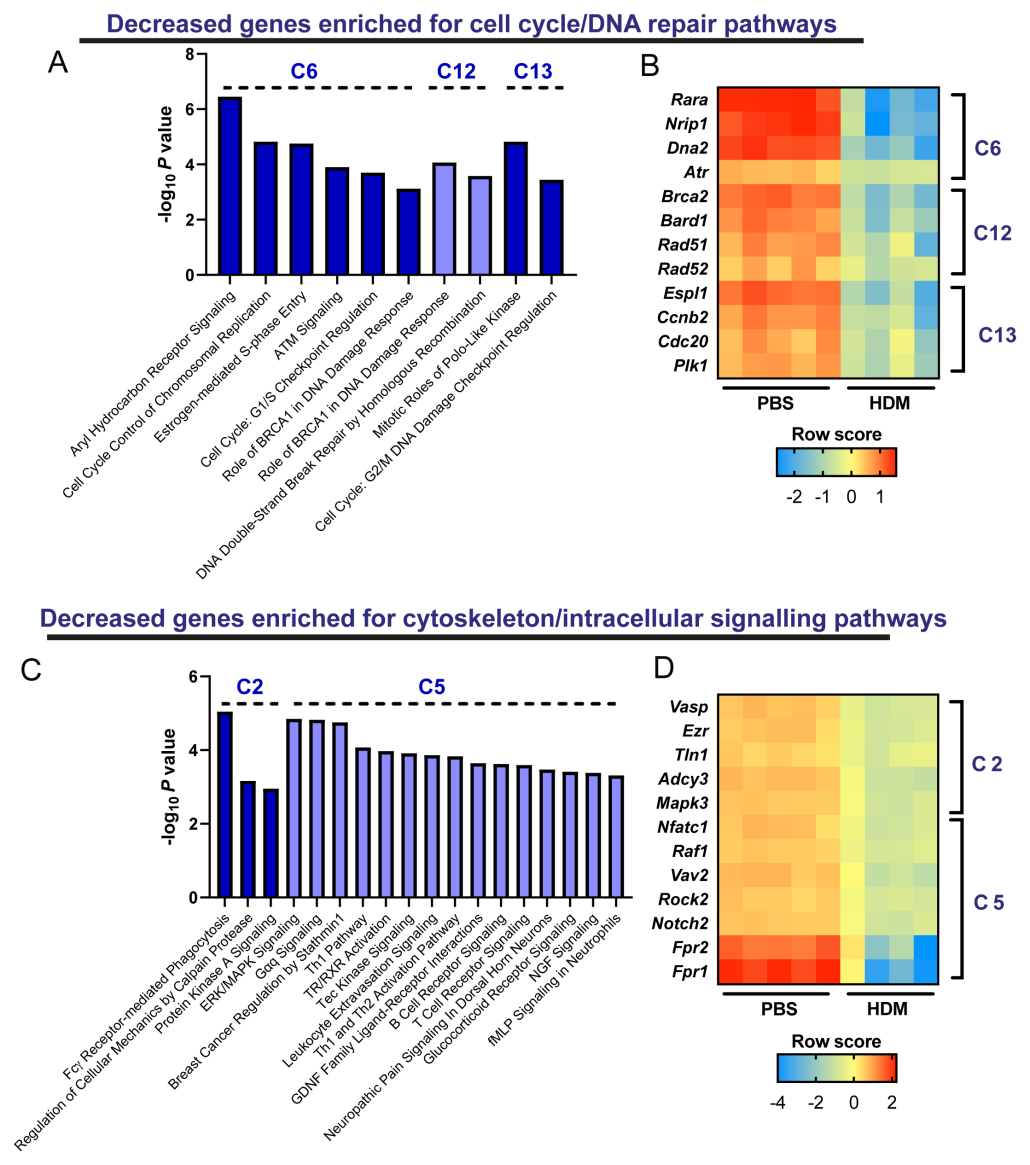
Decreased expression AM ^HDM^ gene clusters enriched for cell cycle, cytoskeletal and intracellular signalling pathways. ( **A**) Highly enriched pathways for clusters of decreased genes in AMs ^HDM^ with pathway annotations related to cell cycle and DNA replication and repair. ( **B**) Expression of representative genes for clusters and pathway annotations shown in ( **A**). ( **C**) Highly enriched pathways for clusters of decreased genes in AMs ^HDM^ with pathway annotations related to cytoskeletal functions and intracellular signalling cascades. ( **D**) Expression of representative genes for clusters and pathway annotations shown in ( **C**). Bar plots show canonical pathway names and inverse enrichment Fisher’s exact *P* values. Heatmaps show row-centred gene expression values (N=5 mice, PBS; N=4 mice, HDM).

Among decreased gene clusters, three (clusters C6, C12 and C13) contained genes highly enriched for pathways related to cell cycle progression and DNA replication and repair, including ‘Mitotic Roles of Polo-Like Kinase’ and ‘Role of BRCA1 in DNA Damage Response’ ( Figure 2A). Specifically, downregulation of DNA repair genes, such as *Atr*, *Rad51* and *Rad52*, and cell cycle genes, such as *Plk1* and genes encoding several cyclins and cyclin-dependent kinases, in AMs ^HDM^ was apparent from these clusters ( Figure 2B). The ‘Aryl hydrocarbon receptor signalling’ pathway was also highly enriched for downregulated cluster C6, but this was largely due to the presence of a number of cell cycle and DNA repair related genes in this canonical pathway. These data therefore suggest that gene expression favouring DNA replication, repair and progression through the cell cycle, a key feature of steady state AMs ^7, 39^, is decreased in the bulk AM ^HDM^ population.

The remaining downregulated gene clusters, C2 and C5, showed relatively high expression in control AMs that was reduced in AMs ^HDM^ ( Figure 1F), suggestive of a set of genes likely involved in steady state AM function that are expressed to a lesser degree in AMs ^HDM^. Genes in C2 were enriched for pathways related to interactions with, and signalling via, the actin cytoskeleton: ‘Fcγ-Receptor-mediated Phagocytosis in Monocytes and Macrophages’ and ‘Regulation of Cellular Mechanics by Calpain Protease’, driven by downregulation of genes encoding actin-associated proteins, such as *Tln1*, *Ezr* and *Vasp* ( Figure 2C, Figure 2D). C5 was also enriched for intracellular signalling pathway genes, such as *Notch2*, *Raf1*, *Vav2* and *Rock1*, resulting in several pathway annotations related to signalling in leukocytes and other cell types, such as ‘T cell receptor signalling’, ‘Leukocyte extravasation signalling’ and ‘Tec kinase signalling’ ( Figure 2C, Figure 2D). Downregulation of both formyl peptide receptor genes ( *Fpr1* and *Fpr2*) in AMs ^HDM^ along with these cell signalling genes resulted in significant over-representation of the ‘fMLP Signaling in Neutrophils’ pathway in C5, suggesting that AM responsiveness to formyl peptide receptor ligands may be reduced during AAD.

Collectively, these data suggested that steady state processes in AMs, such as cell cycle progression, cytoskeletal functions and innate sensing of certain pro-inflammatory stimuli, may be disrupted during allergic airway inflammation.

**AMs ^HDM^ downregulate a subset of genes involved in innate immunity**

**Figure 3.**
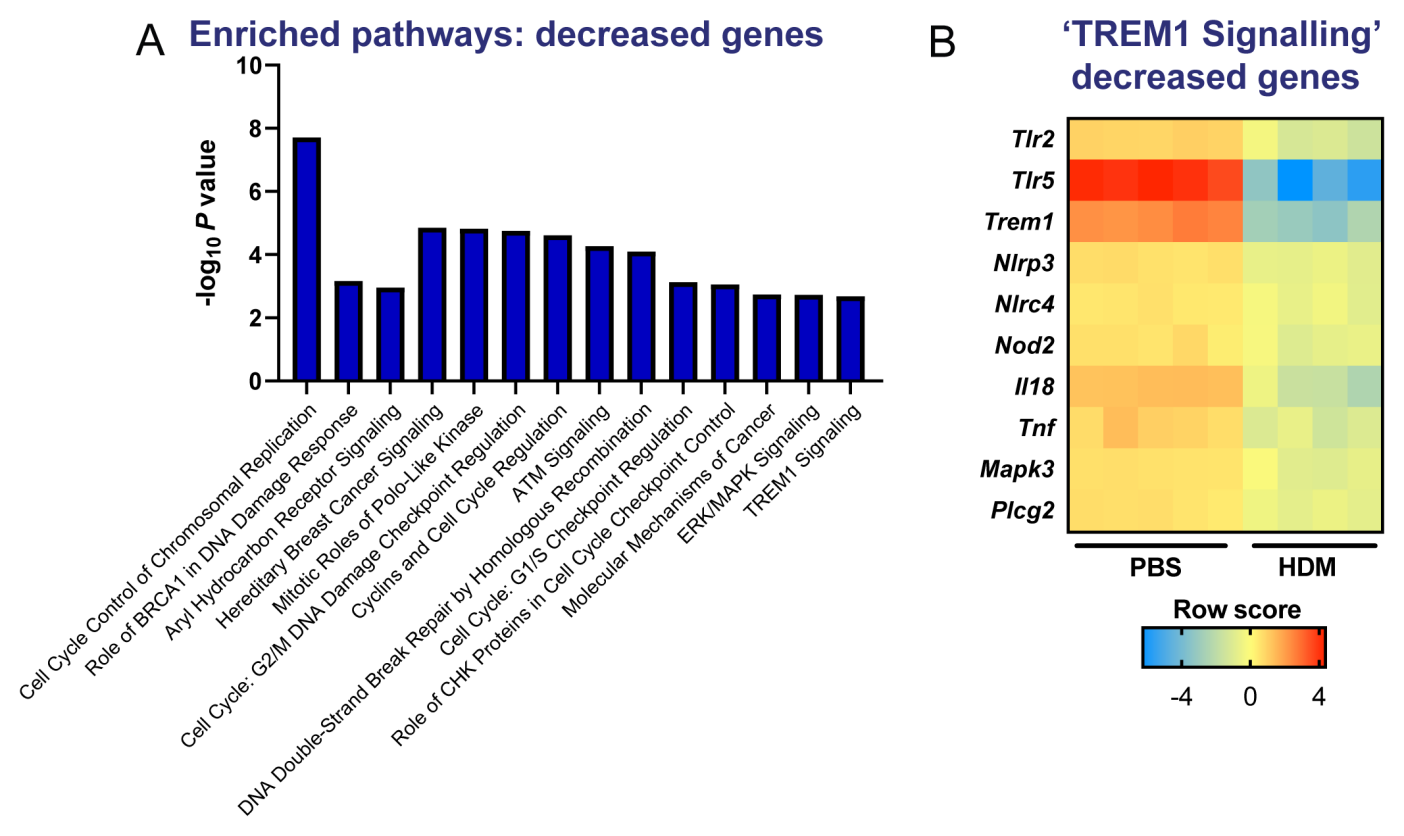
Decreased expression of a subset of innate immune function genes in AMs ^HDM^. ( **A**) Highly enriched pathways for total decreased genes in AMs ^HDM^ ( **B**) Decreased expression of a subset of ‘TREM1 Signalling’ pathway genes in AMs ^HDM^ compared to control AMs. Bar plot shows canonical pathway names and inverse enrichment Fisher’s exact *P* values. Heatmap shows row-centred gene expression values (N=5 mice, PBS; N=4 mice, HDM).

The downregulation of formyl peptide receptors observed in C5 raised the question of whether additional innate immune pathways showed evidence of downregulation in AMs ^HDM^ that were not obvious from the cluster-based analysis. To assess this, pathway analysis was repeated on all downregulated genes together. The results of this analysis were highly dominated by the previously noted decreases in cell cycle and DNA repair-related pathways, which topped the list of most enriched pathways ( Figure 3A). However, the innate immunity-related pathway ‘TREM1 signalling’ was also highly enriched among total downregulated genes, due to significantly reduced expression of genes encoding pattern recognition/inflammasome molecules such as *Tlr2*, *Tlr5*, *Trem1* and *Nlrp3* ( Figure 3B) and the cytokines *Il18* and *Tnf*. **

Thus, despite existing in a highly pro-inflammatory environment, AMs ^HDM^ displayed reduced expression of a number of innate immunity-related genes, consistent with the notion that specific aspects of their protective antimicrobial immune functions are suboptimal during AAD ^40^.

### AMs ^HDM^ display increased expression of antigen presentation and oxidative metabolism genes

**Figure 4.**
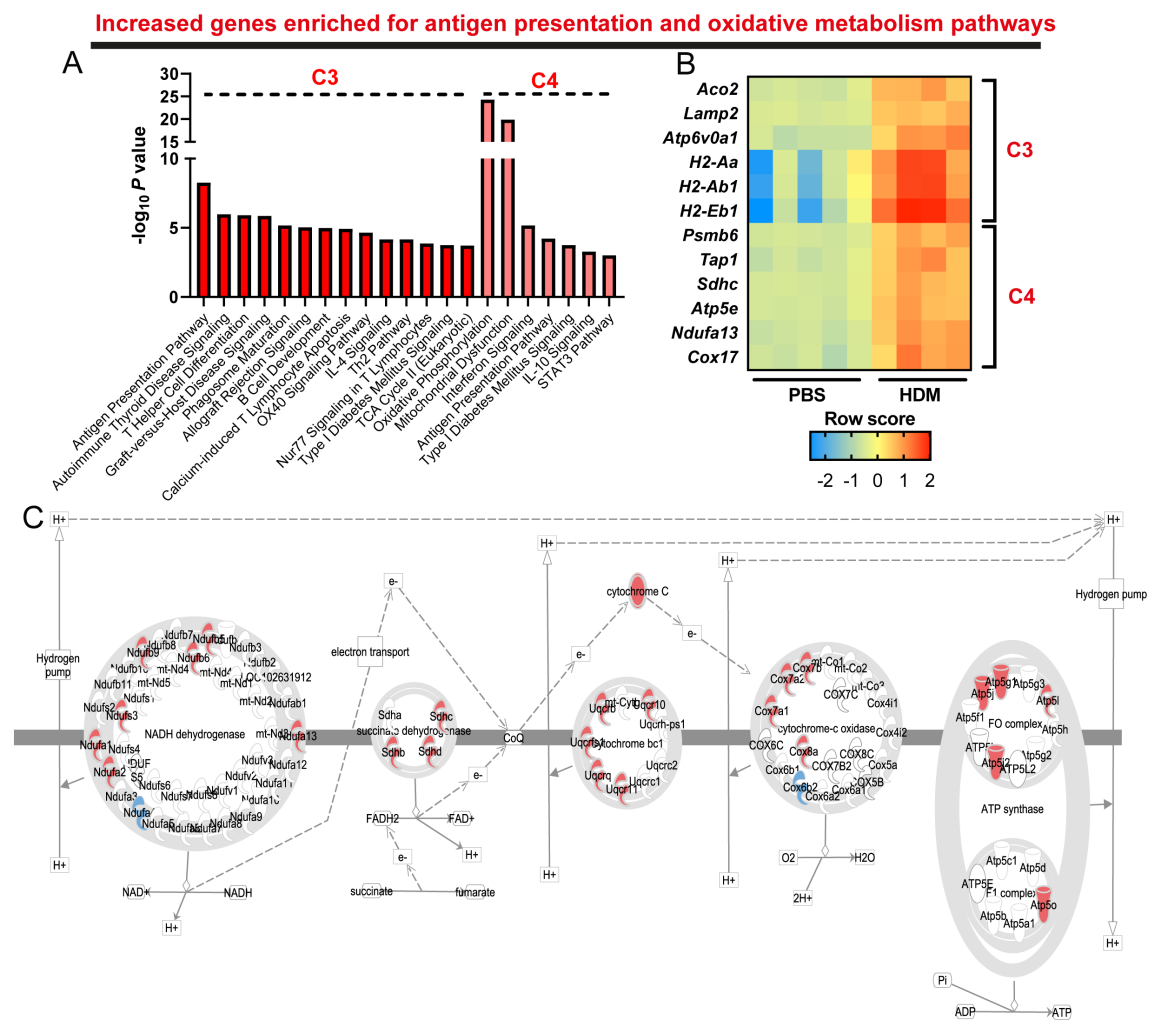
Increased expression AM ^HDM^ gene clusters enriched for antigen presentation and oxidative metabolism pathways. ( **A**) Highly enriched pathways for clusters of increased genes in AMs ^HDM^ with pathway annotations related to antigen presentation and oxidative metabolism. ( **B**) Expression of representative genes for clusters and pathway annotations shown in ( **A**). ( **C**) Pathway diagram for ‘Oxidative Phosphorylation’ with AM ^HDM^ differential gene expression overlaid. Significantly increased genes are shown in red and decreased genes in blue (FDR-adjusted *P* <0.01, fold change ≥2). Bar plot shows canonical pathway names and inverse enrichment Fisher’s exact *P* values. Heatmap shows row-centred gene expression values (N=5 mice, PBS; N=4 mice, HDM).

Upregulated gene clusters C3 and C4 showed moderate to high relative expression in control AMs which was increased in those from HDM-treated mice ( Figure 1F), consistent with constitutively expressed genes that are further elevated during allergic inflammation. Accordingly, these clusters were enriched for pathways related to antigen presentation (e.g. ‘Antigen Presentation Pathway’ and ‘T Helper Cell Differentiation’, Figure 4A), due to upregulation of antigen processing genes, such as *Atp6v0a1*, *Psmb6, Lamp2* and *Tap1*, and a number of class II major histocompatibility complex (MHC) genes *,* including *H2-Aa*, *H2-Ab1* and *H2-Eb1* ( Figure 4B). These data are consistent with previous reports of increased surface MHC-II expression on AMs during HDM-driven allergic inflammation ^41^ and suggest that the capacity for AMs to process antigen and present it on surface MHC molecules increases during AAD.

C4 was also highly enriched for pathways related to oxidative metabolism, specifically ‘oxidative phosphorylation’ and ‘mitochondrial dysfunction’, owing to several electron transport chain (ETC) genes, such as *Atp5e*, *Cox17* and *Ndufa13*, being upregulated in AMs ^HDM^ ( Figure 4B). Indeed, several constituents of all ETC subunits were upregulated at the gene expression level in AMs ^HDM ^( Figure 4C). C3 was also enriched for the ‘TCA Cycle II (Eukaryotic)’ pathway, due in part to upregulation of *Aco2* (aconitase 2), which catalyses the second step of the tricarboxylic acid cycle ( Figure 4A, Figure 4B). These data suggest that the capacity for energy generation by oxidative mitochondrial metabolism is increased in AMs ^HDM^, possibly reflecting a transcriptional shift to overcome the relative metabolic quiescence observed in steady state AMs ^15^.

### AMs ^HDM^ display increased expression of *Aldh1a2* and related small molecule metabolism genes

**Figure 5.**
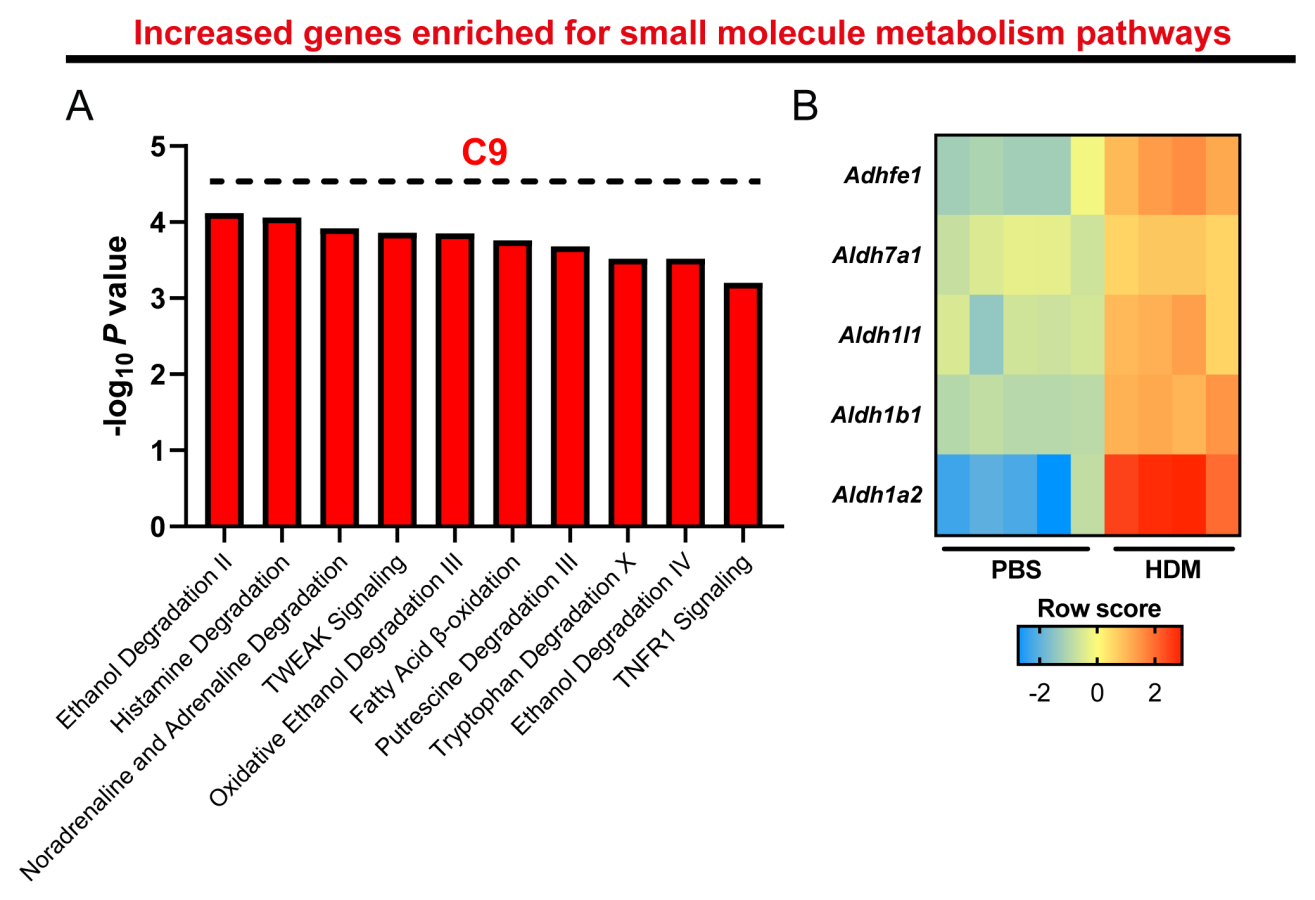
Increased expression AM ^HDM^ genes enriched for small molecule metabolism pathways. ( **A**) Highly enriched small molecule metabolism pathways in cluster C9 of increased genes in AMs ^HDM^. ( **B**) Expression of representative genes for the pathway annotations shown in ( **A**). Bar plot shows canonical pathway names and inverse enrichment Fisher’s exact *P* values. Heatmap shows row-centred gene expression values (N=5 mice, PBS; N=4 mice, HDM).

Cluster C9 was unique among DEG clusters in being enriched for several pathways of small molecule catabolism, including ‘Histamine Degradation’ and various ethanol degradation pathways ( Figure 5A). These enrichments were driven by increased expression of genes encoding aldehyde and alcohol dehydrogenase enzymes in AMs ^HDM^ compared to control AMs ( Figure 5B). Notable among these genes was *Aldh1a2*, encoding retinaldehyde dehydrogenase 2 (RALDH2), which generates the immunoregulatory molecule retinoic acid, which has been implicated in tolerogenic mechanisms of AMs in the context of allergic inflammation ^22^.

Thus, in the context of substantial allergic inflammation, gene expression of a key immunoregulatory enzyme, RALDH2, is increased in AMs, which could potentially indicate a regulatory role for AM-derived retinoic acid during active allergic inflammation.

### Increased expression of inflammation and tissue remodelling genes in AMs ^HDM^

**Figure 6.**
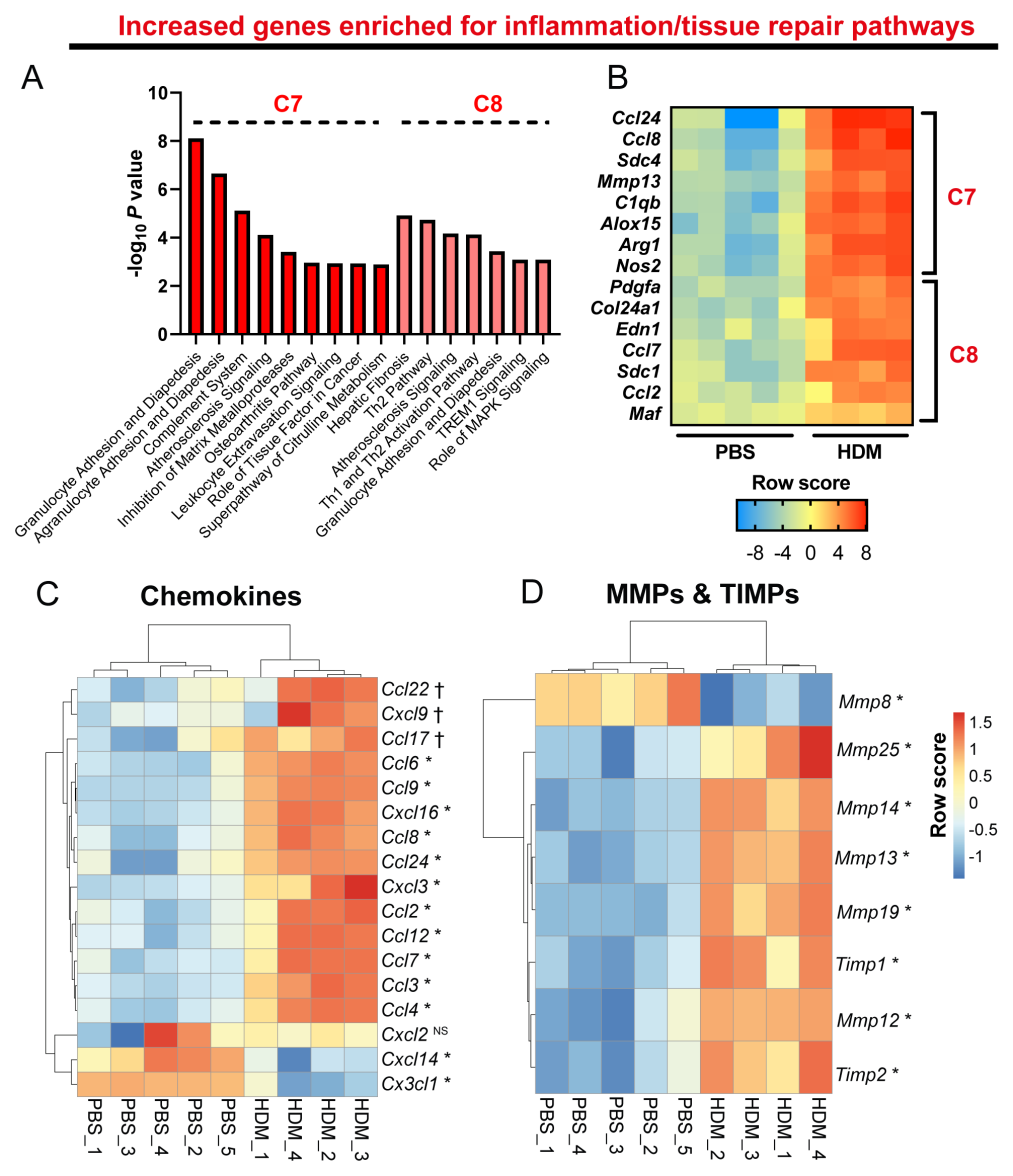
Inflammation- and tissue repair-associated gene expression in AMs ^HDM^. ( **A**) Highly enriched pathways for clusters of increased genes in AMs ^HDM^ with pathway annotations related to inflammatory cell recruitment and tissue repair/remodelling. ( **B**) Expression of representative genes for clusters and pathway annotations shown in A. ( **C**– **D**) Hierarchical clustering of all chemokine (C) and MMP and TIMP (D) genes in all AM samples. Bar plots show canonical pathway names and inverse enrichment Fisher’s exact *P* values. Heatmaps show row-centred gene expression values. Symbols in ( **C**, **D**) refer to results of bulk differential expression analysis by unpaired moderated t-test on all genes: *, *p* < 0.01 after FDR correction; †, *p* < 0.05 before FDR correction; NS, *p* > 0.05 before FDR correction. N=5 mice, PBS; N=4 mice, HDM.

Clusters C7 and C8 were the most strikingly upregulated DEG clusters in AMs ^HDM^ compared to controls, with little to no detectable signal in control AMs ( Figure 1F). These clusters were highly enriched for pathways associated with inflammation and cell recruitment, namely ‘Granulocyte Adhesion and Diapedesis’, ‘Agranulocyte Adhesion and Diapedesis’ and ‘Leukocyte Extravasation Signaling’ ( Figure 6A). These pathway annotations were principally due to the presence of a number of chemokine genes in these highly upregulated gene clusters, including *Ccl2*, *Ccl7*, *Ccl8* and *Ccl24* ( Figure 6B). Clusters C7 and C8 were also enriched for pathways related to ECM remodelling, including ‘Hepatic Cell Fibrosis and Stellate Cell Activation’ and ‘Inhibition of Matrix Metalloproteinases’, due to upregulated genes such as *Mmp13*, *Pdgfa* and syndecans *Sdc1* and *Sdc4* in AMs ^HDM^ ( Figure 6A, B). Of note, AMs ^HDM^ displayed increased expression of *Edn1* in cluster C8, encoding endothelin-1, which has previously been identified as an epithelial-derived mediator of airway remodelling in the context of hyperactivity of the Smad2 signalling pathway ^42^ and is a potent bronchoconstrictor in asthmatics ^43^. The ‘Superpathway of Citrulline Metabolism’ pathway was also highly enriched in C7, owing to upregulation of the competing arginine metabolising enzyme genes *Nos2* and *Arg1* in bulk AMs ^HDM^ ( Figure 6A, B), as we have observed previously by quantitative PCR in a similar HDM AAD model ^41^.

Since the strikingly upregulated C7 and C8 clusters were enriched for genes related to inflammatory cell recruitment and ECM remodelling, chemokine, matrix metalloproteinase (MMP) and tissue inhibitor of metalloproteinase (TIMP) gene expression changes were examined in the whole dataset. Of the chemokine genes detected in AMs, the majority showed increased expression in AMs ^HDM^ as compared to control AMs, including chemokines associated with recruitment of T cells ( *Ccl8*, *Ccl17*, *Ccl22*), monocytes ( *Ccl6*, *Ccl9*, *Ccl2*, *Ccl7*, *Ccl12*) and eosinophils ( *Ccl24*; Figure 6C). Not all chemokines were upregulated in AMs ^HDM^; the neutrophil chemokine *Cxcl2* showed comparable expression to control AMs and expression of *Cxcl14* and *Cx3cl1* was reduced compared to controls ( Figure 6C). Thus, AMs ^HDM^ showed selectively heightened gene expression of a subset of pro-inflammatory chemokines.

Among MMP genes detectable in AMs, all but *Mmp8* displayed significantly greater expression in AMs ^HDM^ than control AMs ( Figure 6D), suggestive of an increased capacity of AMs to contribute to MMP-dependent ECM reorganisation during HDM-driven AAD. Expression of *Timp1* and *Timp2*, which encode regulatory TIMPs, was also increased in AMs ^HDM^ compared to controls ( Figure 6D).

Collectively, these data highlight a gene expression profile of AMs ^HDM ^suggestive of a contribution to inflammatory leukocyte recruitment and ECM remodelling - two key components of AAD pathogenesis.

### Gene expression patterns of AMs ^HDM^ suggest incorporation of multiple cytokine signals from the allergic airway environment

Global transcriptomic analysis of gene clusters showed substantial differences in expression of genes related to diverse functions in AMs present during HDM-driven AAD, compared to those at steady state. Since the phenotype of both embryonic- and adult monocyte-derived AMs is controlled by cytokines in the airway microenvironment ^5, 6, 44, 45^, we sought to identify likely cytokine signals controlling gene expression in AMs during HDM-driven AAD.

**Figure 7.**
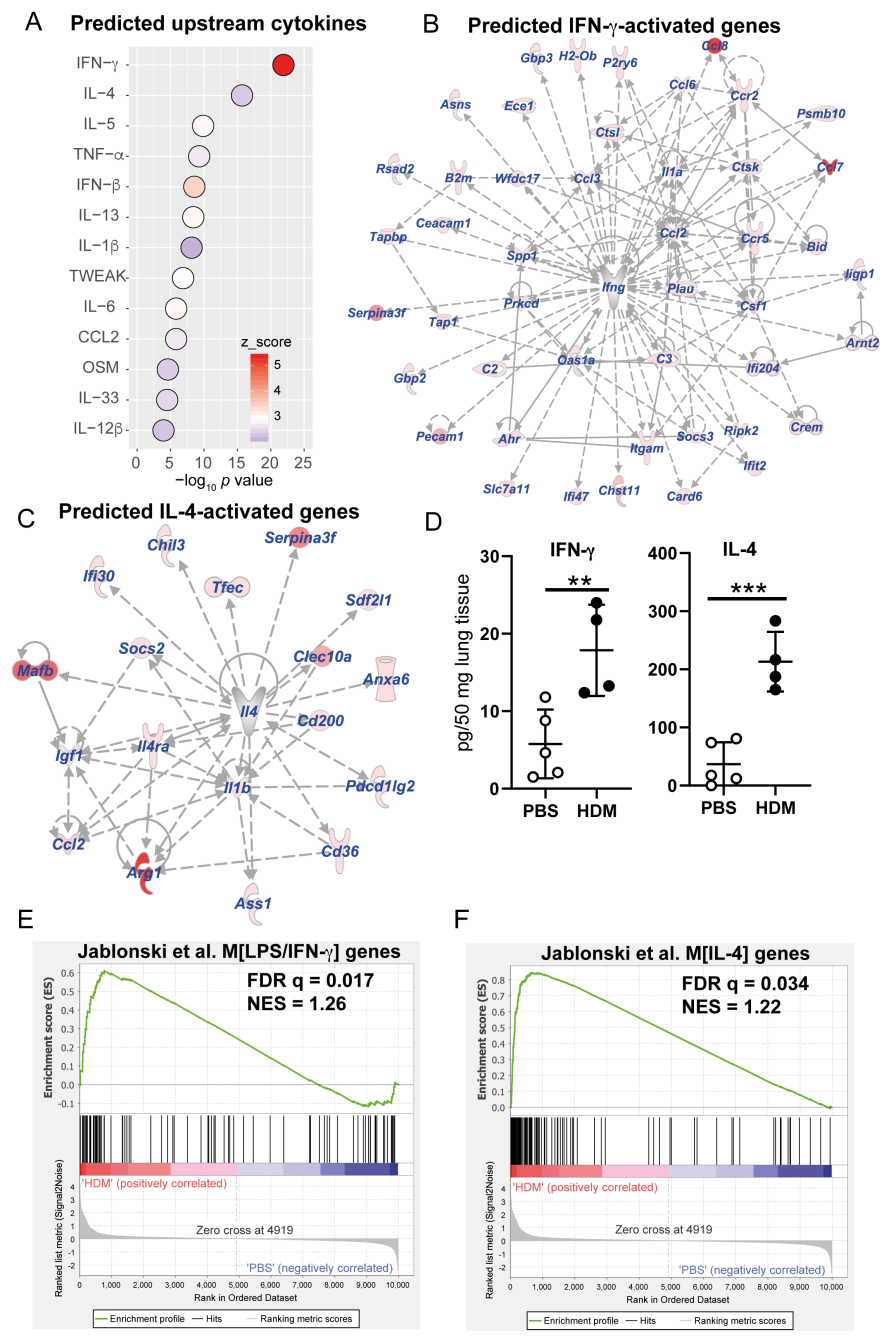
Predicted cytokine regulators of gene expression in AMs ^HDM^ ( **A**) Dot plots showing results of upstream cytokine and growth factor analysis based on differentially expressed genes in AMs ^HDM^. The top regulators with positive activation z scores and highest inverse Fisher’s exact enrichment *P* values are shown. ( **B**, **C**) Network visualisation of genes with an expression pattern in AMs ^HDM^ supporting the predicted activation of IFN-γ ( **B**) and IL-4 ( **C**) in ( **A**). Intensity of red colour is proportional to the fold change in AMs ^HDM^. Arrows indicate literature-reported positive regulation of expression or transcription from the Ingenuity Knowledge Base. ( **D**) Concentration of IFN-γ and IL-4 in homogenised lung tissue as determined by ELISA. ( **E**, **F**) Gene set enrichment analysis for gene sets uniquely upregulated in murine bone marrow-derived macrophages stimulated *in vitro* with LPS and IFN-γ (E, 82 genes) or IL-4 (F, 111 genes), tested for enrichment in AMs ^HDM^ compared to control AMs. False discovery rate q values and normalised enrichment scores are shown. Upstream regulator dot plot shows both inverse *p* values and positive activation z scores of predictions. Cytokine concentration scatter plots show means, standard deviations and individual replicate values, with unpaired student’s t-tests between groups. **, *P* <0.01; ***, *P* <0.001 (N=5 mice, PBS; N=4 mice, HDM).

To this end, total DEGs were subjected to Ingenuity upstream regulator analysis, limiting results to cytokines and growth factors. Of the upstream regulators with a positive predicted activation Z score, IFN-γ and IL-4 were the most significantly predicted to be activated in AMs ^HDM^ ( Figure 7A). Visualisation of the DEGs that supporting these predictions, along with the reported positive regulatory interactions between these genes, revealed distinct IFN-γ- and IL-4-inducible gene networks in AMs ^HDM^ ( Figure 7B, C). Genes did not overlap between the two networks, with the exception of *Ccl2* ( Figure 7B, C), which can be upregulated in macrophages by both IFN-γ and IL-4 ^46^. Other predicted activated upstream regulators included the other type 2 cytokines, IL-5 and IL-13, consistent with a likely major influence of the type 2-dominated environment of the allergic airway on AMs ^HDM^, as well as several known macrophage activators, such as TNF-α, IFN-β and IL-1β ( Figure 7A).

These predictions suggested that the transcriptomic profile observed in total AMs ^HDM^ was the result of the concerted action of several pro-inflammatory cytokines in the allergic lung, including IFN-γ and IL-4. Accordingly, both IFN-γ and IL-4 were present at increased concentrations in lungs of HDM-treated mice than PBS treated controls, with around 10 times more IL-4 detected than IFN-γ after HDM treatment ( Figure 7D). This lung cytokine profile supported the possibility of both IFN-γ and IL-4 shaping AM gene expression during HDM-driven AAD. See *Underlying data* for ELISA output values ^37^.

Finally, since upstream cytokine analysis was based on all literature reported interactions, not limited to macrophages, we validated these results using published data from a controlled model system designed to look specifically at classical (LPS/IFN-γ) and alternative (IL-4) activation responses of macrophages. Lists of upregulated genes after *in vitro* polarisation of murine bone marrow-derived macrophages with either LPS and IFN-γ or IL-4 reported by Jablonksi and colleagues ^35^ were filtered to remove genes upregulated by both treatments and obtain LPS/IFN-γ and IL-4 macrophage gene signatures, enrichment of which was then examined in the AM ^HDM^ transcriptome by gene set enrichment analysis. Both M(LPS/IFN-γ) and M(IL-4) signatures (82 and 111 genes, respectively) were significantly enriched in AMs ^HDM^ compared to control AMs ( Figure 7E), providing further evidence that IFN-γ and IL-4 are both key influencers of AM gene expression during HDM-driven AAD.

Thus, AMs ^HDM^ show altered regulation of diverse gene sets, suggestive of regulation by multiple cytokine signals in the allergic lung.

## Discussion

AMs appear to have a complex role in the immunology of AAD. Both protective and pathogenic roles have been attributed to AMs in the context of AAD ^19– 21, 23^, suggestive of heterogeneity and/or functional plasticity of AMs in the allergic lung. A number of studies have described altered expression of disease-associated genes and proteins in asthmatics and murine AAD models by targeted methods such as flow cytometry, quantitative PCR and immunohistochemistry ^26– 29^. However, no studies to date have examined AM gene expression globally in a clinically relevant mouse model of airway allergen inhalation. Our transcriptomic analysis highlighted several distinct gene expression signatures in the total AM population present following repeated HDM challenge that differed from that in control AMs, overall suggestive of increased inflammatory effector function but decreases in certain steady state AM processes.

Chemotaxis of inflammatory cells to the lung and remodelling of the stromal cells and ECM of the airway wall are key pathobiological features of asthma, which involve the coordinated action of several cell populations ^1^. It is therefore notable that the genes with the most strikingly increased expression in AMs ^HDM^ were enriched for chemokines associated with allergic inflammation, MMPs associated with ECM reorganisation ^47^ and other genes involved in tissue repair and remodelling.

A diverse array of chemokine genes were upregulated in AMs ^HDM^, including chemoattractants of both Th2 cells ( *Ccl17* ^48^ and *Ccl8* ^49^) and eosinophils ( *Ccl24* ^50^), suggestive of a role for AMs in bringing key effector cells into the lung in response to HDM exposure. AMs ^HDM^ also exhibited increased expression of several major agonists of CCR2 and CCR1 ( *Ccl2*, *Ccl7*, *Ccl12*, *Ccl3*, *Ccl4*, *Ccl6* and *Ccl9*) ^51^. Since both CCR2 and CCR1 mediate monocyte recruitment into the lung ^51, 52^, these results indicate that the distinct AMs present in established AAD may act to further shape the mononuclear phagocyte compartment of the allergic lung by promoting accumulation of monocyte-derived cells, which are key local drivers of allergic inflammation ^20, 53^. Indeed, AMs have previously been identified as the major producers of CCL6 during IL-13-driven type 2 lung inflammation, production of which was required for maximal airway inflammation and remodelling ^54^. MMP activity has also been proposed to facilitate migration of inflammatory cells into the airspaces ^55^. It can therefore be speculated that AM-derived chemokines and MMPs cooperate to locally promote inflammation during established AAD. We were interested to observe that *Mmp8*, unlike other MMP genes, was decreased in AMs ^HDM^ as compared to control AMs. This suggests that complex changes might occur to the ECM degradation capabilities of AMs during HDM-driven AAD that warrant further study.

Collectively, these inflammation and tissue repair/remodelling gene expression signatures strongly suggest an active contribution of AMs to the pathogenesis of AAD following repeated HDM exposure and a shift away from their relatively immunologically quiescent steady state phenotype.

AMs ^HDM^ displayed increased expression of a large number of genes related to antigen processing and presentation, consistent with the ability of cytokines elevated in the HDM-allergic airway such as IFN-γ and IL-4 to upregulate MHC-II on macrophages ^41, 56^. The likely consequences of increased antigen processing/presentation genes by AMs ^HDM^ are not clear, since although AMs readily internalise HDM upon inhalation, they are sessile cells and do not carry antigen to draining lymph nodes ^53, 57^. Increased expression of MHC-II and related genes may instead reflect potential for local interactions between inflammatory AMs ^HDM^ and CD4 T cells. Indeed, we have observed co-localisation of CD4 T cells and CD11c ^+^ mononuclear phagocytes, the majority of which are Siglec F ^+^ AMs, during established HDM-driven AAD ^41^.

Despite a clear increase in antigen processing and presentation genes, two downregulated gene clusters in AMs ^HDM^ were enriched for genes related to cytoskeletal functions and related signalling pathways. Downregulation of genes related to such cytoskeleton and intracellular signalling pathways have also been observed in AMs following LPS inhalation ^39^, suggesting that these gene expression changes may reflect common features of AM responses to distinct pro-inflammatory stimuli. Since actin polymerisation signalling is important for macrophage phagocytosis ^58^, decreased expression of genes involved in actin cytoskeleton signalling could be indicative of decreased phagocytic capacity in AMs ^HDM^, as has been observed in human asthmatics ^59^. Indeed, AMs ^HDM^ also showed a significant decrease in mRNA expression for the apoptotic cell uptake receptor AXL, suggestive of a possible decrease in the capacity for efferocytosis using this receptor. However, *Axl* expression in these cells remained relatively high so it is unclear whether this decrease would have functional impact. Decreased *AXL* mRNA has also been observed in human asthmatic AMs and is associated with impaired efferocytosis of apoptotic cells in these patients ^38^, while AXL knockout mice display impaired resolution of lung inflammation following influenza infection ^60^, consistent with an important function of this receptor in maintaining and restoring immune homeostasis in the airway. The likely functional consequences of a decrease in expression of *Axl* and phagocytosis-related cytoskeletal genes, but increased antigen presentation and processing genes in AMs ^HDM^ are not clear from these data alone. Functional assays of the capacity of AMs ^HDM ^to phagocytose different cell types, such as apoptotic leukocytes and pathogenic bacteria, will shed more light on these results.

Increased pro-inflammatory gene expression in AMs ^HDM^ was accompanied by increased expression of genes related to oxidative metabolism, particularly those encoding mitochondrial ETC subunits, suggesting that capacity for ATP generation in AMs by oxidative phosphorylation is increased during HDM-driven AAD. Similar upregulation of electron transport chain genes has previously been reported during the acute response of AMs, but not IMs, to LPS *in vivo* ^39^, suggestive of a common feature of activated AMs in several inflammatory settings. Moreover, increased electron transport chain gene expression has also been demonstrated in peritoneal macrophages following IL-4Rα-dependent activation during nematode infection ^32^. Notably, IL-4Rα-dependent peritoneal macrophages also displayed increased expression of *Ccl8* and *Ccl24* ^32^ during anti-nematode immunity and polarisation of GM-CSF-differentiated human monocyte-derived macrophages with IL-4 increased expression of both oxidative metabolism genes and the chemokine *CCL22* ^61^. Collectively, these findings suggest that an association exists between oxidative metabolic gene expression and chemokine production by macrophages activated in type 2 immune settings, particularly since increased oxidative phosphorylation is a central feature of macrophages activated alternatively by type 2 cytokines such as IL-4 ^61, 62^. Such a relationship between macrophage activation and metabolism is unlikely to be limited to oxidative phosphorylation, since inhibition of glycolysis has been shown to reduce the IL-4-induced gene expression in murine AMs that was enhanced upon *ex vivo* culture ^15^. Although gene expression is not a perfect surrogate for cellular metabolic phenotype these data prompt more detailed analysis of AM ^HDM^ metabolism to reveal whether these cells indeed break their reported steady state metabolic quiescence ^15^ and whether metabolic reprogramming regulates their production of inflammatory mediators in the allergic airway.

Not all gene expression differences in AMs ^HDM^ were pro-inflammatory. AMs ^HDM^ had reduced expression of the antimicrobial inflammatory cytokine genes *Tnf* and *Il18*, as well as *Tlr2*, which was previously reported in AMs ^HDM^ at the level of cell surface expression and associated with impaired antibacterial immunity ^40^. AMs ^HDM^ also showed reduced expression of the positive regulator of TLR signalling, *Trem1*, as observed previously for neutrophils in a similar HDM model ^40^, along with a substantial decrease in *Tlr5*, which is essential for certain antibacterial responses in AMs ^63^. Notably, TLR5 has also been shown to recognise flagellin present in HDM extracts to promote AAD, suggesting that AM ^HDM^ sensing of HDM could be altered in our AAD model, although the specific role of AMs in TLR5-dependent sensing of flagellin in HDM extracts is not known ^64^. Our transcriptional data therefore support the notion that certain antimicrobial functions in AMs are impaired during allergic airway inflammation ^14, 40^.

We also observed increased expression of *Aldh1a2*, encoding the retinoic acid biosynthetic enzyme RALDH2, in AMs ^HDM^. Since AM-derived retinoic acid has been proposed to contribute to their tolerogenic capacity ^22^, increased RALDH2 expression by AMs ^HDM^ could present a mechanism of protective immune regulation by these cells even during established allergic inflammation. An active immune regulatory role of AMs during AAD and not just prior to allergic sensitisation would go some way to explain why AM depletion enhances HDM-driven AAD even when cells are depleted when allergic inflammation is already established ^21^. However, it is important to note that retinoic acid function is context-dependent and can be pro-inflammatory in certain settings ^65^.

Several of the gene expression changes in AMs ^HDM^ were consistent with IL-4Rα-dependent, alternatively-activated macrophages, as expected given the type 2 immune environment of the HDM-allergic lung. However, gene expression signatures in AMs ^HDM^ were also suggestive of upstream influence from other cytokines, notably the classical macrophage activator IFN-γ. This mixed transcriptional response supports the idea that the classical/alternative macrophage activation paradigm fails to capture the phenotype of tissue macrophages *in vivo*, where they are exposed to several cytokine signals simultaneously ^66^, and that a spectrum of activation states exists based on combinatorial effects of different signals ^67^. The increased expression of both IL-4 and IFN-γ-induced genes in AMs ^HDM^ is interesting with these ideas in mind, since IFN-γ and IL-4 are considered to be opposing and antagonistic activators of macrophages ^68^. However, a subset of genes induced by these cytokines are relatively refractory to cross-inhibition by the opposing cytokine, indicating that mixed IFN-γ/IL-4 activation states are possible in macrophages ^68^. One interpretation of the mixed gene expression profile observed in bulk AMs ^HDM^ is therefore that these cells are a relatively homogeneous population that integrates signals from several cytokines present in the allergic airway, including type 2 cytokines and IFN-γ, to achieve an intermediate activation state. Alternatively, several AM subsets may exist during HDM-driven AAD, some with an IL-4-dominated transcriptional profile and others with greater expression of classical macrophage activation genes, which could conceivably contribute to distinct protective and/or pathogenic AM functions in the allergic lung. Single cell transcriptomic and immunophenotypic analysis of AMs ^HDM^ will be valuable in untangling this.

Heterogeneity within the bulk AM ^HDM^ population may reflect cells of different ontogeny, since AMs derived from circulating inflammatory monocytes have been characterised both after primary influenza infection and bleomycin-induced lung injury in mouse models and shown to be transcriptionally and functionally distinct from prenatally-derived long term-resident AMs ^69, 70^. It is therefore possible that the bulk AM ^HDM^ population observed in our model comprises AMs resident throughout the treatment period and one or more additional subpopulations derived from inflammatory monocytes. Decreased expression of a large number of cell cycle genes in AMs ^HDM^ relative to control AMs could result from incorporation of monocyte-derived macrophages into the total AM population, since resident AMs undergo steady state proliferation in the absence of inflammation ^7^ and express high levels of cell cycle genes relative to IMs and pulmonary monocytes ^39^, so expression of cell cycle genes could conceivably be diluted within the bulk AM ^HDM^ population by monocyte-derived inflammatory AMs that have exited the cell cycle. Alternatively, cell cycle gene expression in resident AMs could be decreased during HDM-driven AAD. A recent study showed cell cycle gene expression to be decreased in murine AMs present after self-limiting pneumococcal infection ^71^, suggesting that this may be a common feature of inflammation-experienced AMs.

Further studies specifically labelling and characterising monocyte-derived versus resident AMs in the HDM AAD model will be necessary to determine the extent to which gene expression observed in AMs ^HDM^ reflects plasticity of resident AMs or local differentiation and instruction of infiltrating monocytes by signals in the allergic airway environment. If multiple AM ^HDM^ subpopulations of different ontogeny exist, it will be important to determine the protective and/or pathogenic functions of each subset in AAD, given that monocyte-derived AMs, rather than tissue-resident AMs, have been identified as drivers of bleomycin-induced pulmonary fibrosis ^69^ and mediators of prolonged innate protection against *Streptococcus pneumoniae* infection following resolution of influenza infection ^70^. Furthermore, it will be important to determine how long distinct AM ^HDM^ transcriptional signatures and phenotypes persist following cessation of allergen exposure, given the importance of AMs in resolution of HDM-driven AAD ^21^.

Collectively, our data identify substantial gene expression changes in AMs during experimental AAD compared to those present in naïve mice in the steady state. Although the HDM inhalation mouse model recapitulates many of the hallmark features of allergic asthma ^30, 72^, several differences exist between human and murine lungs and it will therefore be important to examine these and other molecular signatures in AMs from human asthmatics. Nonetheless, our AM ^HDM^ gene expression provide useful insights into potential widespread changes in AM behaviour in the allergic lung, including reductions in steady state functions and increases in those related to AAD pathogenesis, likely governed by a combination of cytokine signals. We believe that these findings will prompt and support further study of AMs in experimental AAD and human asthma.

## Data availability

### Underlying data

NCBI Gene Expression Omnibus: Transcriptomic analysis of airway macrophages from a murine experimental allergic airway disease model. Accession number GSE148590; https://identifiers.org/geo:GSE148590.

This accession contains the raw and processed data files from the RNA-seq study. A list of differentially expressed genes and their associated expression clusters is provided as extended data.

Open Science Framework: Supporting material for 'Transcriptomic analysis reveals diverse gene expression changes in airway macrophages during experimental allergic airway disease'. https://doi.org/10.17605/OSF.IO/8Q3S7 ^37^.

This project contains the following underlying data:

- ELISA_absorbance_values. (Raw ELISA absorbance values supporting IFN-γ and IL-4 concentrations shown in Figure 7D.)
- Flow_cytometry_files. (Raw flow cytometry files supporting AM and eosinophil numbers shown in Figure 1B.)

### Extended data

Open Science Framework: Supporting material for 'Transcriptomic analysis reveals diverse gene expression changes in airway macrophages during experimental allergic airway disease'. https://doi.org/10.17605/OSF.IO/8Q3S7 ^37^.

Folder ‘GSEA_input’ contains input gene lists used for gene set enrichment analysis shown in Figure 7E.

Folder ‘Extended_data’ contains the airway macrophage counts for Figure 1B, airway eosinophil counts for Figure 7D, and the list of differentially expressed genes and their clusters.

### Reporting guidelines

Open Science Framework: ARRIVE checklist for Transcriptomic analysis reveals diverse gene expression changes in airway macrophages during experimental allergic airway disease'. https://doi.org/https://doi.org/10.17605/OSF.IO/8Q3S7 ^37^.

Data are available under the terms of the Creative Commons Attribution 4.0 International license (CC-BY 4.0).
