# Supplementary material for: Transcriptomic analysis reveals diverse gene expression changes in airway macrophages during experimental allergic airway disease
Source: Wellcome Open Res. 2020 Jun 22;5:101. Originally published 2020 May 26. [Version 2] doi: 10.12688/wellcomeopenres.15875.2 (PMC7309452; doi:10.12688/wellcomeopenres.15875.2)
Supplement: Peer review history

# Peer review: Reviewer response for version 1

**DOI:** 10.21956/wellcomeopenres.17412.r38860

**Published:** 2020-06-15

**Authors:** Padraic Fallon

**Affiliations:** 1 School of Medicine, Trinity College Dublin, Dublin, Ireland

This paper delivers an original and in-depth profile of gene expression in airway macrophages (AW), using bulk RNA sequencing, in the lungs of mice in the steady state and during an allergic airway disease (AAD) model. The authors are leaders in this arena and the study is underpinned by a robust HDM AAD model and sequencing analysis. The attention to detail in the comprehensive Methods section is notable.

 The paper is well written and highly informative. It raises new insight on multiple aspect of functions of AM in allergic lung tissue, including cell heterogenicity, innate immunity ( *Tlr5*) and metabolism ( *Aldh1a2).*

 This paper provides an essential data set that will support future characterisation of mouse AM, including single cell sequencing, in the normal and allergic lung and in other states of pulmonary disease. 

 Minor comments.

1. In the abstract (Methods) include the specific cell surface profile ("CD11c+ Siglec F+ CD64+ CD45+ SSChi") of AM. 
2. Fig 1. The addition of BAL IFN-g and IL-4 expressing CD4 cells in BAL would illustrate the Th1 and Th2 cellular dynamics of this specific HDM AAD model. The inclusion is not essential; but would extend discussions on Figure 7, including panel 7D, on AM in the prevailing lung cytokine environs. 
3. Figures 2B and 2D, 3B, 4B, 5B, 6B. "Expression of representative genes for clusters and pathway annotations shown in A".
  These are not clear and would benefit from presentation in a different graphic format. 
  Data are mean (n= 4 or n=5) showing statistical differences or just inferred relative expression? For example, Fig 3B, *Tlr5* is evidently different between PBS v HDM, but are the others genes?

Is the work clearly and accurately presented and does it cite the current literature?

Yes

If applicable, is the statistical analysis and its interpretation appropriate?

Yes

Are all the source data underlying the results available to ensure full reproducibility?

Yes

Is the study design appropriate and is the work technically sound?

Yes

Are the conclusions drawn adequately supported by the results?

Yes

Are sufficient details of methods and analysis provided to allow replication by others?

Yes

Reviewer Expertise:

Immunology. Mouse models of inflammatory diseases, including pulmonary inflammation.

I confirm that I have read this submission and believe that I have an appropriate level of expertise to confirm that it is of an acceptable scientific standard.

# Response

**Published:** 2020-06-16

**Authors:** William Branchett

**Affiliations:** Imperial College London, UK

We are grateful for this thoughtful and thorough review of our manuscript. We have revised the manuscript in response to the feedback provided as detailed below.

In the abstract (Methods) include the specific cell surface profile ("CD11c+ Siglec F+ CD64+ CD45+ SSChi") of AM. 

*We have now included this in the Methods section of the Abstract to more clearly describe the study*

Fig 1. The addition of BAL IFN-g and IL-4 expressing CD4 cells in BAL would illustrate the Th1 and Th2 cellular dynamics of this specific HDM AAD model. The inclusion is not essential; but would extend discussions on Figure 7, including panel 7D, on AM in the prevailing lung cytokine environs. 

*Thank you for this suggestion. It is indeed likely that CD4 T cells are providing the cytokines that instruct gene expression in AMs. Unfortunately, we did not acquire these data at the time of analysis of our HDM model. We agree that this is non-essential for the manuscript as it is, but understanding the sources of cytokines that drive different gene expression profiles will be an important component of future studies of AMs in AAD.*

Figures 2B and 2D, 3B, 4B, 5B, 6B. "Expression of representative genes for clusters and pathway annotations shown in A". These are not clear and would benefit from presentation in a different graphic format. Data are mean (n= 4 or n=5) showing statistical differences or just inferred relative expression? For example, Fig 3B, *Tlr5* is evidently different between PBS v HDM, but are the others genes?

*We agree that the presentation of these data in the original manuscript was misleading, as the log2-transformed normalised expression values were shown, resulting in very small visible distances between points and small to invisible error bars. This was also highlighted as a point for improvement by Reviewer 1. To better reflect the variance within groups and the differences between groups, we have now replaced these dot plots with row-centred heatmaps.*

We thank the reviewer again for their constructive feedback.

# Peer review: Reviewer response for version 1

**DOI:** 10.21956/wellcomeopenres.17412.r38859

**Published:** 2020-06-10

**Authors:** Sylvia Knapp, Asma Farhat, Mariem Radhouani

**Affiliations:** 1 Laboratory of Infection Biology, Medical University Vienna, Vienna, Austria

In their study “Transcriptomic analysis reveals diverse gene expression changes in airway macrophages during experimental allergic airway disease”, Branchett *et al*. performed bulk RNA-seq on sorted AMs from the BAL of HDM or PBS challenged mice. 

 The data presented by the authors demonstrates a shift in the gene expression signature of AMs upon HDM challenge, with a decrease in steady state associated genes and an increase in genes related to inflammatory cell recruitment and ECM remodeling. 

 The authors further validated upstream key regulators of AMs gene expression during AAD (IFN-g and IL-4) which opens possibilities of future experiments to functionally characterize AMs in AAD.

 Altogether, the article is very clear and well written. The conclusions drawn by the authors are adequate and correspond to the data shown.

 Overall, we think this study is interesting and relevant and deserves to be indexed in Wellcome Open Research. While the study is descriptive in nature, it still provides substantial information and is a good resource for further investigation of the complex role of AMs in allergic asthma.

**Comments:**

 No major comments.

 Minor comments:

- Figure 2B, 2D, 3B, 4B, 5B and 6B: The plots showing the expression of representative genes for the different clusters are unusually depicted. This could be optimized, for example by using a heatmap. 
- Figure 3B: wording of figure legend (underlined) “ Expression values of expression in AMs” could be changed.
- Figure 4C: The pathway diagram could be improved by changing the font size/style to make it clearer and more readable, as the way it is now, a lot of the components of the diagram are overlapping making it very hard to decipher. 
- Figure 7 B and C: the font size could be increased to make the networks clearer.

Is the work clearly and accurately presented and does it cite the current literature?

Yes

If applicable, is the statistical analysis and its interpretation appropriate?

Yes

Are all the source data underlying the results available to ensure full reproducibility?

Yes

Is the study design appropriate and is the work technically sound?

Yes

Are the conclusions drawn adequately supported by the results?

Yes

Are sufficient details of methods and analysis provided to allow replication by others?

Yes

Reviewer Expertise:

Immunology, macrophage biology, lung inflammation.

We confirm that we have read this submission and believe that we have an appropriate level of expertise to confirm that it is of an acceptable scientific standard.

# Response

**Published:** 2020-06-16

**Authors:** William Branchett

**Affiliations:** Imperial College London, UK

We are grateful for this thorough review of our manuscript. We have implemented the suggestions as far as possible and feel that this has improved the presentation of the data.

In response to the specific comments:

Figure 2B, 2D, 3B, 4B, 5B and 6B: The plots showing the expression of representative genes for the different clusters are unusually depicted. This could be optimized, for example by using a heatmap

*All dot plots showing representative gene changes have been replaced with row-centred heat maps to better highlight the magnitude of differential expression between groups.*

Figure 3B: wording of figure legend (underlined) “ Expression values of expression in AMs” could be changed.

*We apologise for overlooking this small typing error, which has now been corrected.*

Figure 4C: The pathway diagram could be improved by changing the font size/style to make it clearer and more readable, as the way it is now, a lot of the components of the diagram are overlapping making it very hard to decipher. 

*We are unfortunately unable to make this particular figure element any larger and changes to font sizes would result in further overlap between labels. The figure is provided in high resolution to allow zooming while viewing online. Since the main intention of this panel was to highlight that transcriptional changes were observed across the whole electron transport chain, we hope that it is still informative as it is. The expression values of the individual genes are readily available in the Extended Data table of clustered differentially expressed genes supporting this manuscript.*

Figure 7 B and C: the font size could be increased to make the networks clearer.

*We have increased the sizes of the two network panels by 20% to improve readability.*

We thank the reviewers again for their constructive feedback.
